# Adsorption and inhibition mechanism of pyrazole derivatives on carbon steel: combined electrochemical, surface, and DFT/MD study

**DOI:** 10.1039/d6ra00508j

**Published:** 2026-03-19

**Authors:** A. Barrahi, A. Marzaq, R. Saddik, S. Tighadouini, F. Benhiba, M. El Faydy, B. Dikici, M. Abdallah, I. Warad, A. Zarrouk

**Affiliations:** a Laboratory of Molecular Spectroscopy Modelling, Materials, Nanomaterials, Water and Environment, CERNE2D, Faculty of Sciences, Mohammed V University in Rabat Morocco asmaabarrahi@gmail.com azarrouk@gmail.com a.zarrouk@um5r.ac.ma +212-697474666; b Laboratory of Organic Chemistry, Materials, Electrochemistry and Environment, Faculty of Sciences Ain Chock, Hassan II University BP 5366 Casablanca Morocco; c Higher Institute of Nursing Professions and Health Techniques of Casablanca, Annex Settat Casablanca Morocco; d Laboratory of Applied Chemistry and Environment (LCAE), Faculty of Sciences, Mohammed First University 60000 Oujda Morocco; e Ataturk University, Department of Mechanical Engineering 25240 Erzurum Turkey; f Chemistry Department, Faculty of Science, Benha University Benha Egypt; g Department of Chemistry, AN-Najah National University P. O. Box 7 Nablus Palestine

## Abstract

In the present work, the effectiveness of two pyrazole derivatives, *i.e.* (*Z*)-1-(1-benzyl-5-methyl-1*H*-pyrazol-3-yl)-3-(3-ethylpyrazin-2-yl)-3-hydroxyprop-2-en-1-one (PMB) and (*Z*)-1-(1-benzyl-5-methyl-1*H*-pyrazol-3-yl)-3-hydroxy-3-(1*H*-imidazol-1-yl)prop-2-en-1-one (PMI), as corrosion inhibitors for carbon steel in an acidic medium was investigated using various experimental methodologies such as potentiodynamic polarization (PDP), electrochemical impedance spectroscopy (EIS), surface analysis, and theoretical simulation by density functional theory (DFT) and molecular dynamics (MD). The inhibition mechanism was also performed to comprehend the inhibition mechanism. The results showed that the inhibition efficiencies of PMB and PMI increase with concentration and decrease with temperature, reaching a maximum efficiency of 95.3% for PMB and 82.5% for PMI at 303 K. PDP results reveal that the two inhibitors exhibit a mixed-type inhibition process, which inhibits both the anodic dissolution of metal and the cathodic reduction reaction. The EIS results reveal that higher polarization resistance (*R*_p_) and lower double layer capacitance (*C*_dl_) are observed due to the adsorption of molecules and protective film formation. AFM and SEM-EDS evidence a more homogeneous and less deteriorated surface, whereas contact angle measurements and UV-vis results corroborate the enhancement in hydrophobicity and the presence of inhibitor–interface interactions. Furthermore, the theoretical results are corroborated with experimental results and provide a good explanation of the adsorption pattern of PMB and PMI chemicals, which undergo a type of chemisorption according to the Langmuir adsorption model.

## Abstract 2

PMB and PMI pyrazole derivatives were investigated as corrosion inhibitors for carbon steel in acidic medium using PDP, EIS, surface analysis, DFT and MD simulations. Both compounds showed strong adsorption on the steel surface and significant inhibition efficiency.

## Graphical abstract

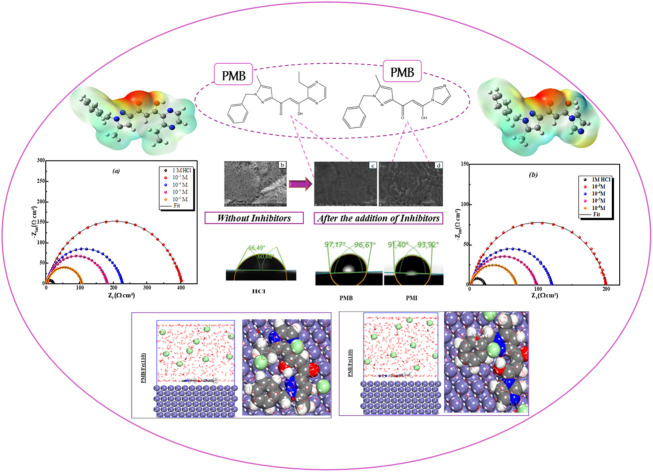

## 1. Introduction

Carbon steel is recognized for its superior mechanical properties, broad temperature range of use, and low cost.^1,2^ Its use may cause corrosion, at least in part due to its chemically reactive nature.^3,4^ Strong acids can greatly speed up this process, serving to shorten the life span of the materials and develop other types of corrosion. Then, it is necessary to measure the corrosion for carbon steel as precisely as possible, particularly under acidic conditions, which are among the most aggressive. For many of these processes, such as pickling, cleaning, descaling, or acidifying, this profile is replicated. The most commonly used pickling agents are HCl, HNO_3_, and H_2_SO_4_.^5,6^ To reduce this degradation, various solutions have been envisaged (modification of metallurgical properties, protective coatings, electrochemical protection, or the use of inhibitors).^7,8^ Of these techniques, the use of synthetic corrosion inhibitors is most widely used due to their effectiveness, low cost, high efficiency, and versatility.

‘Organic’ molecules with polar groups, especially nitrogen-, oxygen- or sulfur-containing species within them, as well as π-electron-rich heterocyclics, have been shown to retard corrosion.^9–11^ The inhibition effect is attributed to their adsorption on the metal surface, leading to a pronounced decrease in the dissolution process and thus retarding interfacial corrosion at the metal/solution interface. N-heterocyclic–pyrazole hybrids have emerged as the best corrosion inhibitors for steel in acidic environments. This is because of their heterocyclic structure, which has several nitrogen atoms that can donate their lone pairs to strongly adsorb the metal surface through coordination bonding and π-interaction with the d-orbitals of iron.^12^ Besides that, these hybrids are quite environmentally friendly, have low toxicity compared to conventional inorganic inhibitors, and can be easily synthesized with various substituents to maximize their inhibition performance.^13^ In the present study, we deliberately selected two N-heterocyclic hybrids sharing the same pyrazole ring to enable a focused comparison, yet differentiated by the heterocyclic substituent linked to the pyrazole ring: one bearing an imidazole ring (a five-membered diazole) and the other an ethylpyrazin-2-yl group (a six-membered diazine linked *via* an ethyl). Even though both substituents have the same number of nitrogen atoms besides those in pyrazole, the difference in structure is due to the changes in the fundamental aspects of ring form, electron property, and flexibility in shape. The imidazole substituent is a small, highly basic five-membered ring with high π-donor and coordination abilities through two nitrogen atoms; thus, it can adsorb tightly, possibly in a flat manner, and produce a strong film. On the other hand, the ethylpyrazin-2-yl part involves a basic six-membered ring with extended conjugation. Also, the ethyl chain is carried by the pyrazine ring, which brings more steric bulk and rotational freedom and changes the balance between hydrophobicity and hydrophilicity. Such differences, although the content of nitrogen is almost the same, can greatly affect the electron density distribution, the most preferred adsorption orientation, and the interaction strength with the steel surface, as well as the compactness and durability of the protective film. By comparing these closely related yet structurally distinct hybrids, this work provides novel insights into unveiling the role of ring size/aromatic character/spacer effects in inhibition performance variations within the pyrazole ring, relationships that are still underexplored for this specific pair of heterocycles, thus helping in the development of the rational design of new, highly efficient and environmentally friendly corrosion inhibitors for acidic industrial environments. Furthermore, a comparison with results published in the scientific literature,^13–18^ obtained under similar experimental conditions, demonstrated that the hybrids studied exhibit significant efficiency (Table 1).

**Table 1:** Comparison of our pyrazoles' performance with literature findings under similar experimental conditions

Inhibitors	Metal	Solution	*η* (%)	Ref.
Ethyl 5-methyl-1-(((4-nitrophenyl)amino)methyl)-1*H*-pyrazole-3-carboxylate	Carbon steel	1 M HCl	87%	13
*N*-((3,5-Dimethyl-1*H*-pyrazol-1-yl)methyl)pyrimidin-2-amine	Mild steel	1 M HCl	91%	14
5-Ethyl-1-methyl-((pyrimidin-2-ylamino)methyl)-1*H*-pyrazole-3-carboxylate	Mild steel	1 M HCl	92%	14
(3,5-Dimethyl-1*H*-pyrazol-1-yl)(4-((4-chlorobenzy-lidene)amino)phenyl)methanone	Carbon steel	1 M HCl	89%	17
*N*-((3,5-Dimethyl-1*H*-pyrazol-1-yl)methyl)-4-nitroaniline	Carbon steel	1 M HCl	95%	18
(*Z*)-1-(1-Benzyl-5-methyl-1*H*-pyrazol-3-yl)-3-(3-ethylpyrazin-2-yl)-3-hydroxyprop-2-en-1-one	Carbon steel	1 M HCl	**95.3%**	**This work**
(*Z*)-1-(1-Benzyl-5-methyl-1*H*-pyrazol-3-yl)-3-hydroxy-3-(1*H*-imidazol-1-yl)prop-2-en-1-one	Carbon steel	1 M HCl	**82.5%**	

The novelty of this work is to investigate the anticorrosion behavior of two newly synthesized pyrazole derivatives, (*Z*)-1-(1-benzyl-5-methyl-1*H*-pyrazol-3-yl)-3-(3-ethylpyrazin-2-yl)-3-hydroxyprop-2-en-1-one (PMB) and (*Z*)-1-(1-benzyl-5-methyl-1*H*-pyrazol-3-yl)-3-hydroxy-3-(1*H*-imidazol-1-yl)prop-2-en-1-one (PMI), on carbon steel in 1 M HCl solution. Their performance was compared by various electrochemical techniques such as potentiodynamic polarization (PDP) and electrochemical impedance spectroscopy (EIS). Concomitantly, surface analytical techniques such as scanning electron microscopy and contact angle measurements were used to investigate their interaction with the metal substrate. These compounds also proved to be PMB > PMI in terms of inhibitory potency, at 95.3% and 95.3%, respectively, according to the obtained results. The adsorption process and thermodynamic parameters have been investigated, indicating that the inhibitors obeyed the Langmuir isotherm model and the film formed on the metal surface contains an inhibitor–metal complex. Furthermore, MD and DFT simulations provide additional insights into the adsorption mechanism, consistent with experimental data.

## 2. Experimental details

### 2.1. Materials and inhibitors

The carbon steel samples used in this study had the following chemical composition: in mass percent C (0.370), Si (0.230), Mn (0.680), S (0.0160), Cr (0.0770), Ni (0.0590), Ti (0.011), Co (0.009), Cu (0.16) and Fe (balance). For the electrochemical experiments, samples with a surface area of 1 cm^2^ were prepared. Before each test, the steel electrode was meticulously polished with silicon carbide (SiC) abrasive discs of various grain sizes to obtain a clean and homogeneous surface. The electrode was then degreased with acetone, washed with distilled water, and dried at room temperature.

**Fig. 1:**
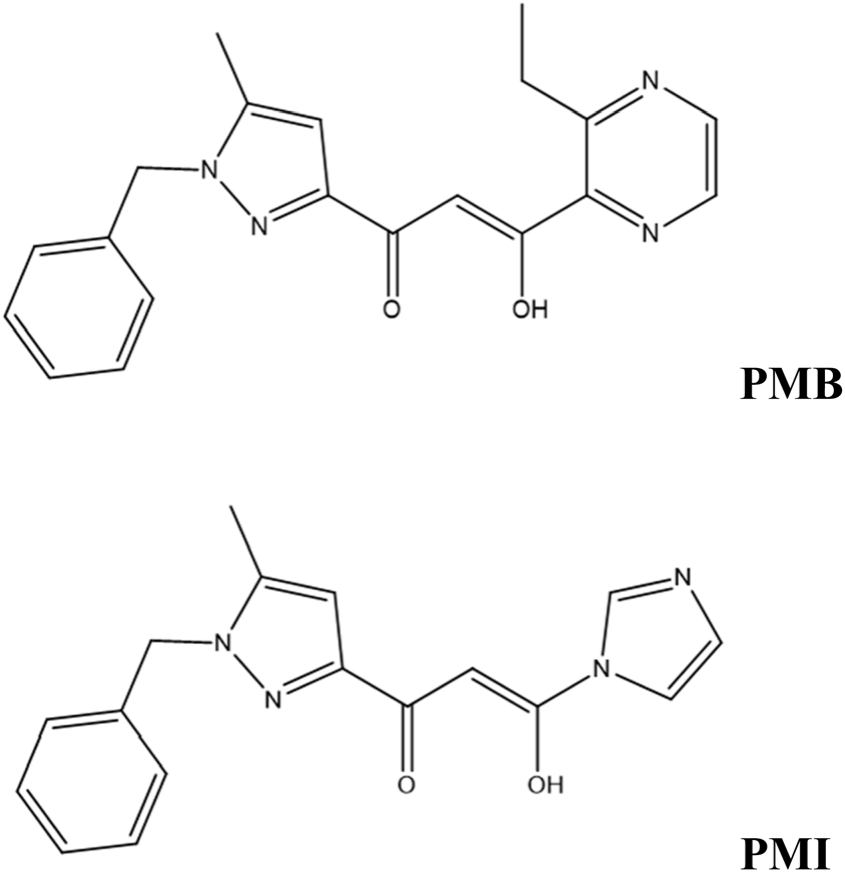
Molecule structures of PMB and PMI.

The electrolyte for the corrosion study was 1 M HCl prepared by diluting 37% hydrochloric acid in distilled water. In addition, four concentrations ranging from 10^−3^ to 10^−6^ M were evaluated. Fig. 1 shows the structure of (*Z*)-1-(1-benzyl-5-methyl-1*H*-pyrazol-3-yl)-3-(3-ethylpyrazin-2-yl)-3-hydroxyprop-2-en-1-one (PMB) and (*Z*)-1-(1-benzyl-5-methyl-1*H*-pyrazol-3-yl)-3-hydroxy-3-(1*H*-imidazol-1-yl)prop-2-en-1-one (PMI).

### 2.2. General procedure for the synthesis of β-keto–enol heterocycles (PMB and PMI)

The synthesis procedure was adjusted according to the previously peported literature.^19,20^ Metallic sodium (15.21 mmol) was suspended in toluene (20 mL), and a solution of the ethyl 1-benzyl-5-methyl-1*H*-pyrazole-3-carboxylate (12.01 mmol) in toluene (25 mL) was added dropwise under stirring. Subsequently, 1-acetylimidazole or the 1-(3-ethylpyrazin-2-yl)ethanone (12.01 mmol), dissolved in toluene (10 mL), was added at 0 °C. The reaction mixture was allowed to warm to room temperature and stirred continuously for 3 days. Upon completion of the reaction, the resulting solid was collected by filtration and washed with toluene. The crude product was dissolved in water and carefully acidified to pH 6 with acetic acid. The mixture was extracted with CH_2_Cl_2_, and the organic layer was dried over anhydrous sodium sulfate and concentrated under reduced pressure. The resulting residue was purified by silica gel column chromatography using CH_2_Cl_2_/MeOH as the eluent to afford compounds PMB and PMI. The structural characterization was performed using FT-IR, ^1^H NMR, and ^13^C NMR spectroscopy, as well as mass spectrometry.

#### 2.2.1 (*Z*)-1-(1-Benzyl-5-methyl-1*H*-pyrazol-3-yl)-3-(3-ethylpyrazin-2-yl)-3-hydroxyprop-2-en-1-one (PMB)

Yield 21%; *R*_f_: 0.24 CH_2_Cl_2_/MeOH 8/2/silica. IR (KBr, cm^−1^): *ν*(OH) = 3478; *ν*(C=O) = 1670; *ν*(enolic C=C) = 1543; ^1^H-NMR (DMSO-d_6_): *δ* 1.45 (d, 3H, CH_3_–pyrz); 2.36 (s, 3H, CH_3_–Pz); 3.33 (q, 2H, CH_3_–CH_2_–pyrz); 5.86 (s, 2H, Ar–CH_2_–Pz); 6.59 (s, 1H, Pz–H); 6.88 (s, 1H, enol, C–H); 7.24–7.36 (m, 5H, Ar); 8.32 (m, 2H, N=CH–pyrz). ^13^C-NMR (DMSO-d_6_): *δ* 10.2 (1C, Pz–CH_3_); 13.34 (1C, CH_3_–pyrz); 28.78 (1C, CH_2_–pyrz); 56.48 (1C, Ar–CH_2_–Pz); 96.18 (1C, enol, C–H); 106.30 (1C, C=CH–Pz); 128.1–137.45 (6C, Ar); 146.34 (1C, N=C–pyrz) 148.23 (1C, N=C–pyrz); 188.9 (1C, C=O); 192.26 (1C, C–OH); MS: *m*/*z*, 350.20 (M + 2H)^+^.

#### 2.2.2 (*Z*)-1-(1-Benzyl-5-methyl-1*H*-pyrazol-3-yl)-3-hydroxy-3-(1*H*-imidazol-1-yl)prop-2-en-1-one (PMI)

Yield 32%; *R*_f_: 0.70 (CH_2_Cl_2_/MeOH 8/2/silica), IR (KBr, cm^−1^): *ν*(OH) = 3478; *ν*(C=O) = 1680; *ν*(enolic C=C) = 1540; ^1^H-NMR (DMSO-d_6_): *δ* 2.14 (s, 3H, Pz–CH_3_); 5.23 (s, 2H, Pz–CH_2_–Ar); 6.37 (s, 1H, enol, C–H); 7.02 (d, 1H, C2H–imid); 7.48 (m, 5H, Ar); 7.75 (d, 1H, C3H–imid). 8.34 (s, 1H, C1H–imid) ^13^C-NMR (DMSO-d_6_): *δ* 12.13 (1C, Pz–CH_3_); 59.49 (2C, Ar–CH_2_–Pz); 83.13 (1C, enol, C–H); 116.1 (2C, C2–Pz and C3–imid); 124.56 (1C, C1–Pz); 127.2–138.34 (6C, Ar–C1,2,3,4,5,6); 128.8 (1C, C3–imid); 129.3 (1C, C2–imid Ar–C3,5); 138.8 (1C, Pz, C–CH_3_); 186.17 (1C, C–OH); 187.15 (1C, C=O); MS: *m*/*z*, 309.12 (M + H)^+^.

### 2.3. Corrosion test

Electrochemical experiments were performed utilizing a Voltalab PGZ-100 potentiostat managed by a computer and linked to the “Voltamaster 4” program. All electrochemical experiments were performed using a typical three-electrode cell. A carbon steel with a 1 cm^2^ surface serves as the working electrode, the platinum auxiliary electrode, and the saturated calomel reference electrode (SCE). To stabilize the cell before testing, the WE was submerged in varied concentrations of test media for 30 minutes at corrosion-free potential (*E*_ocp_).

After determining the corrosion potential *E*_ocp_, electrochemical tests were carried out by automatically shifting the electrode potential from −800 mV to −100 mV relative to *E*_ocp_ at a scan rate of 0.5 mV s^−1^.^21^ The polarization curves were used to derive linear Tafel segments from the anodic and cathodic curves to calculate corrosion current densities (*i*_corr_) at corrosion potential. Furthermore, electrochemical impedance spectroscopy (EIS) studies were performed under identical circumstances as the PDP. The EIS frequencies applied ranged from 10^5^ Hz to 10^−2^ Hz with 10 mV sinusoidal perturbation potential. The impedance data was analyzed and corrected with ZView software.

### 2.4. Surface analysis

#### 2.4.1. UV-visible

The corrosive medium containing optimal concentrations of PMB and PMI inhibitors was evaluated by UV-visible spectrophotometry before and after 24 h immersion in the acidic solution. The experiment in this work was carried out using a Jenway 67 series UV-visible spectrophotometer.

#### 2.4.2. CA and MEB/EDS

The samples were polished with abrasive paper before immersion in HCl solution, with or without 10^−3^ M of PMB and PMI inhibitors. The specimens were polished with abrasive papers and then immersed in HCl solution containing 10^−3^ M of the PMB and the PMI inhibitors. Contact angle values were measured in 6 h immersion in 1 M HCl with PMB and PMI at optimized concentration by Dataphysics OCA 50. Scanning electron microscope images were acquired with a JEOL JSM-IT 100 under a 20 kV acceleration voltage and equipped with an EDS unit. The images of the metal surface were made by an examination at 10 000× magnification, for SEM-EDS analysis was performed at 24 hours. It is interesting to highlight that the results on the electrochemical tests (concentration and temperature effect) behavior of the uninhibited solution determined in this work are in good agreement with those polished by our prior manuscript, since all experiment were performed together with the same apparatus, the same applies to the contact angle and SEM-EDS analysis.^22^

### 2.5. Theoretical study

#### 2.5.1. DFT

The molecular modeling and visualization of the inhibitor compounds under investigation were generated and prepared in the GaussView 6 program. All quantum calculations were then performed with the Gaussian 09 package. The geometries of compounds PMB and PMI in aqueous solution were fully optimized applying Density Functional Theory (DFT), with the B3LYP functional and 6-311+G(d,p) basis set. This tool allows a more realistic description of molecular interaction. The GaussView 6 program was used to see the molecular structures, map the boundary orbitals (HOMO and LUMO), and calculate the related energies. These energies were then used to calculate other important electronic properties, including gap energy (*E*_gap_), ionization potential (*I*), electronic affinity (*A*), molecular hardness (*η*), and electronegativity (*χ*) by utilizing the following formulas:^23,24^

1 *I* = −*E*_HOMO_

2 *A* = −*E*_LOMO_

3 *E*_gap_ = *E*_LOMO_ − *E*_HOMO_

4
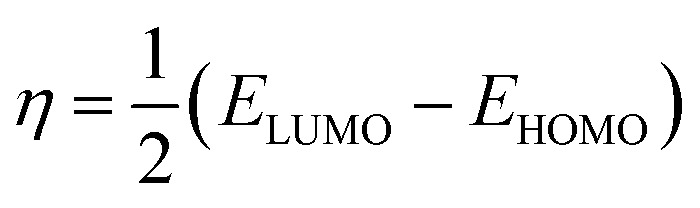
5
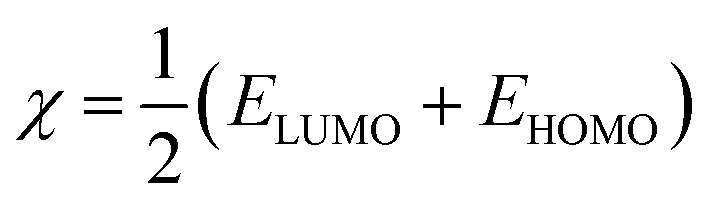
6
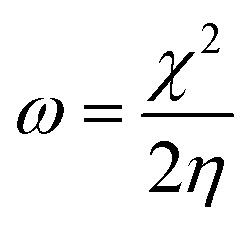
7
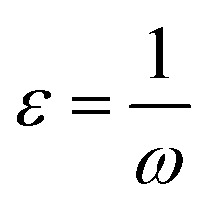


#### 2.5.2. Molecular dynamics simulation

The mechanism of inhibition of the PMB and PMI compounds was elucidated and simulated by molecular dynamics (MD) simulation utilizing well-defined model parameters.^25,26^ In the present simulation, the 9C^−^, 9H_3_O^+^, 491H_2_O, Fe(110), and the inhibitors studied were investigated. In the context of this simulation, MD was performed thanks to the Forcite module incorporated in the Materials Studio 2023 software. The model system was based on the application of periodic boundary conditions in all 3-D. A unit cell with dimensions of 27.30 × 27.30 × 37.13 Å^3^ is employed, representing a supercell with a surface area of 11 × 11 of the metal substrates.

Before the MD, the geometrical structure of the inhibitor compounds was optimized by means of the Generalized Gradient Approximation (GGA) and the Dual Digital with Polarization (DNP) basis of calculation to obtain an energetically stable configuration. The COMPASS III force field was employed throughout the simulation.

The simulation was performed over a total duration of 2000 ps with a time step of 1.0 fs. The evolution of the system was guaranteed under the canonical ensemble (*NVT*), keeping constant the number of particles (*N*), the volume (*V*), and the temperature (*T*). Temperature control was carried out using the Andersen thermostat, which enables efficient regulation *via* stochastic collisions with a virtual thermal bath. The temperature used in all simulations was kept constant at 303 K.^27^

## 3. Finding results

### 3.1. OCP curves

**Fig. 2:**
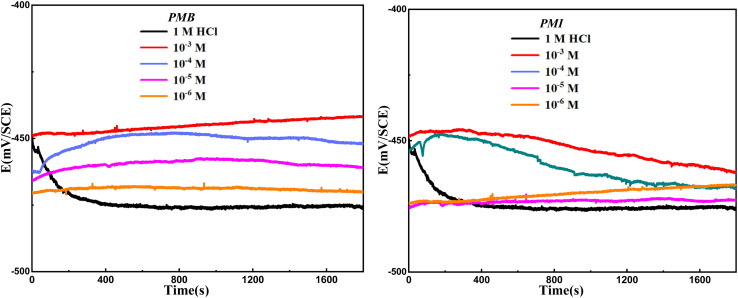
The development of the OCP as a function of time of carbon steel in 1 M HCl without and with different concentrations of PMB and PMI.

The influence of PMB and PMI on the carbon steel corrosion was investigated by measuring the open circuit potential in 1 M HCl. The evolution of the *E*_ocp_ potential as a function of time is often measured before any other electrochemical test, as it does not affect the properties of the sample. In this investigation, the working electrode was submerged in the test medium for 1800 seconds to measure the open circuit potential. As shown in Fig. 2, the curve stabilizes after this period of immersion, suggesting that the carbon steel has achieved stability. It is also noted that the *E*_ocp_ values of the inhibitors shift towards higher positive potentials, which can be attributed to the adsorption of PMI and PMB on the surface of the carbon steel, thus slowing its corrosion.

### 3.2. PDP curves

#### 3.2.1. Effect of concentration

**Fig. 3:**
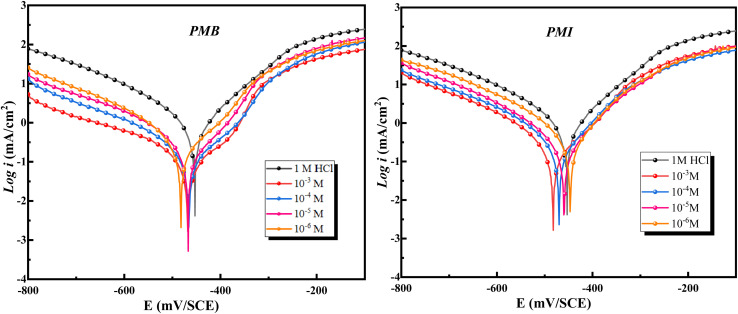
Polarization curves for CS in 1 M HCl without and with the presence of PMB and PMI.

Fig. 3 shows the potentiodynamic polarization curves for carbon steel in 1 M HCl in the absence and the presence of different doses of pyrazole derivatives (PMB and PMI). The addition of PMB and BMI compounds results in a considerable negative change in corrosion potential, indicating their activity as mixed-type inhibitors (maximum shift < 85 mV).^28,29^ The graphs show that pyrazole derivatives do not alter the reaction mechanism but rather reduce the anodic and cathodic current densities, which slow down the steel dissolution and hydrogen reduction processes. Efficiency *η*_p_ (%) improves with concentration, significantly reducing corrosion.

Table 2 shows that corrosion current densities decline with increasing inhibitor concentration, with minimum values of 52.2 µA cm^−2^ for PMB and 192.9 µA cm^−2^ for PMI at 10^−3^ M. The inhibitor efficiency increased with this decrease, reaching 95.3% for PMB and 82.5% for PMI. This is clarified by the fact that the organic compounds PMB and PMI adhere to the metal surface, replacing the water molecules and creating a strong barrier.^30^ This layer prevents proton reduction and metal dissolution in the media by blocking the anodic and cathodic sites. The addition of PMB and PMI changes the *β*_c_ values, indicating that these derivatives have an impact on the kinetics of hydrogen evolution and effectively lessen acid corrosion of carbon steel.^2,31^ Additionally, the change in *β*_a_ values demonstrates that PMB and PMI affect the kinetics of the anodic process. These results demonstrate that the number of active sites on the metal surface is reduced by the formation of a protective film induced by adsorption.^32^ In addition, the oxygen and nitrogen atoms in both PMB and PMI contribute to blocking the steel degradation by producing an Fe–pyrazole derivative complex. The inhibitive action of the pyrazole derivatives is strengthened by the presence of the aromatic ring, which in general favors π–π interactions on the metal surface, and –OH groups easily donate electrons contributing to maintaining a stable protective film. However, there are several reasons for the greater potency of PMB. Unlike imidazole, where one of the nitrogen's is primarily involved in ring stabilization, the pyrazine present in PMB has two aromatic nitrogen's that are ideally positioned to interact with the active sites of the metal. In addition, the ethyl group of PMB acts as an electron donor substituent, increasing the electron density of the ring and strengthening its bonds with the metal.

**Table 2:** Polarization data for CS corrosion in 1 M HCl in the existence and nonexistence of PMB and PMI

	*C* (M)	−*E*_cor_ (mV per SCE)	*i*_cor_ (µA cm^−1^)	*β*_a_ (mV dec^−1^)	−*β*_c_ (mV dec^−1^)	*η*_p_ (%)
HCl	1 M	456.3 ± 5.8	1104.1 ± 4.9	112.8	155.4	—
PMB	10^−3^	467.4 ± 3.7	52.2 ± 2.2	104.0	97.8	**95.3**
	10^−4^	464.1 ± 4.9	87.4 ± 5.3	99.0	98.0	92.1
	10^−5^	466.4 ± 7.2	121.4 ± 3.6	97.2	88.3	89.0
	10^−6^	480.1 ± 4.5	189.4 ± 4.0	110.1	83.6	82.8
PMI	10^−3^	482.2 ± 5.3	192.9 ± 3.1	122.1	83.9	**82.5**
	10^−4^	470.2 ± 8.5	256.9 ± 2.4	101.8	87.4	76.7
	10^−5^	460.5 ± 3.2	341.8 ± 3.3	122.3	90.2	69.0
	10^−6^	446.8 ± 6.6	487.4 ± 4.7	121.8	83.7	55.8

#### 3.2.2. Temperature effect

**Fig. 4:**
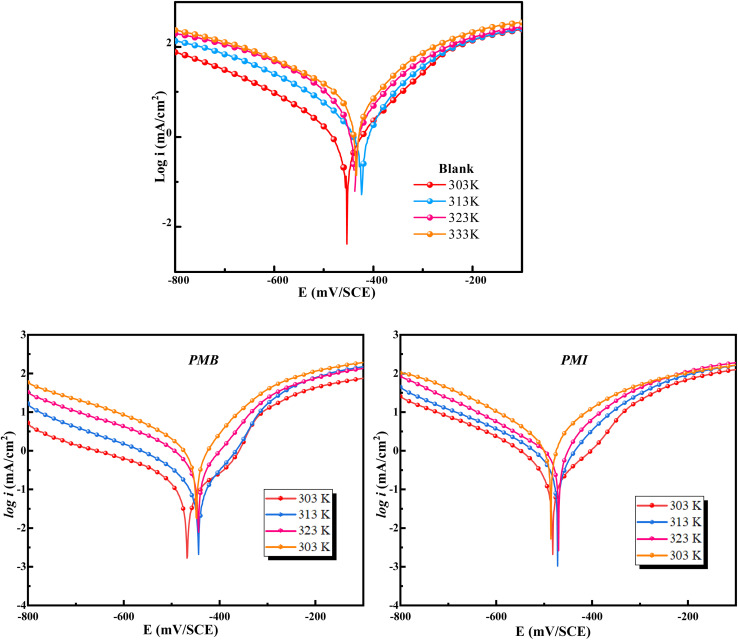
Polarization plots for CS in 1 M HCl solution in the presence and absence of 1 mM of PMB and PMI at temperatures ranging from 303 K to 333 K.

The potentiodynamic polarization (PDP) curves for CS immersed in a 1 M HCl medium at various temperatures, both with and without 10^−3^ M PMB and PMI, are shown in Fig. 4.

Table 3 shows the quantitative information obtained from these curves. According to the data, both inhibited and non-inhibited solutions show an increase in corrosion current (*i*_corr_) with increasing temperature. However, this increase was more pronounced in the uninhibited electrolyte, demonstrating the protective effect of the PMB and PMI compounds. Furthermore, the efficiency of inhibition tended to decrease with increasing temperature. This process can be explained by the gradual desorption of the pyrazole derivative molecules adsorbed on the carbon steel surface.^33^ The substrate becomes more susceptible to the corrosive assault of the acid as a result of the weakening of the adsorptive interactions caused by the increase in thermal energy. Furthermore, despite the temperature increase, the PMB maintains its performance relative to the PMI.

**Table 3:** PDP parameters of CS corrosion in 1 M HCl in the existence and nonexistence of optimum concentrations of PMB and PMI at different concentrations

Milieu	*T* (K)	−*E*_corr_ (mV per SCE)	*i*_corr_ (µA cm^−2^)	*β*_a_ (mV dec^−1^)	−*β*_c_ (mV dec^−1^)	*η*_p_ (%)
1 M HCl	303	456.3 ± 5.8	1104.1 ± 4.9	112.8	155.4	—
	313	423.5 ± 9.0	1477.4 ± 7.8	91.3	131.3	—
	323	436.3 ± 7.0	2254.0 ± 9.0	91.4	117.8	—
	333	433.3 ± 5.0	3944.9 ± 10.0	103.9	134.6	—
PMB	303	467.4 ± 3.7	52.2 ± 2.2	104.0	97.8	95.3
	313	443.5 ± 5.5	92.8 ± 1.7	102.4	77.4	93.7
	323	443.4 ± 3.9	200.1 ± 3.0	72.0	63.2	91.1
	333	448.5 ± 7.1	456.2 ± 5.4	80.5	63.0	88.4
PMI	303	482.2 ± 5.3	192.9 ± 3.1	122.1	83.9	82.5
	313	472.8 ± 2.8	302.2 ± 7.3	98.1	68.7	79.5
	323	471.3 ± 4.2	509.2 ± 5.9	96.0	61.0	77.4
	333	485.5 ± 6.3	975.9 ± 7.3	95.0	61.3	75.3

To better understand how temperature affects the corrosion process, Arrhenius and transition state diagrams were utilized to evaluate the activation properties of carbon steel corrosion in 1 M HCl at different temperatures.

**Fig. 5:**
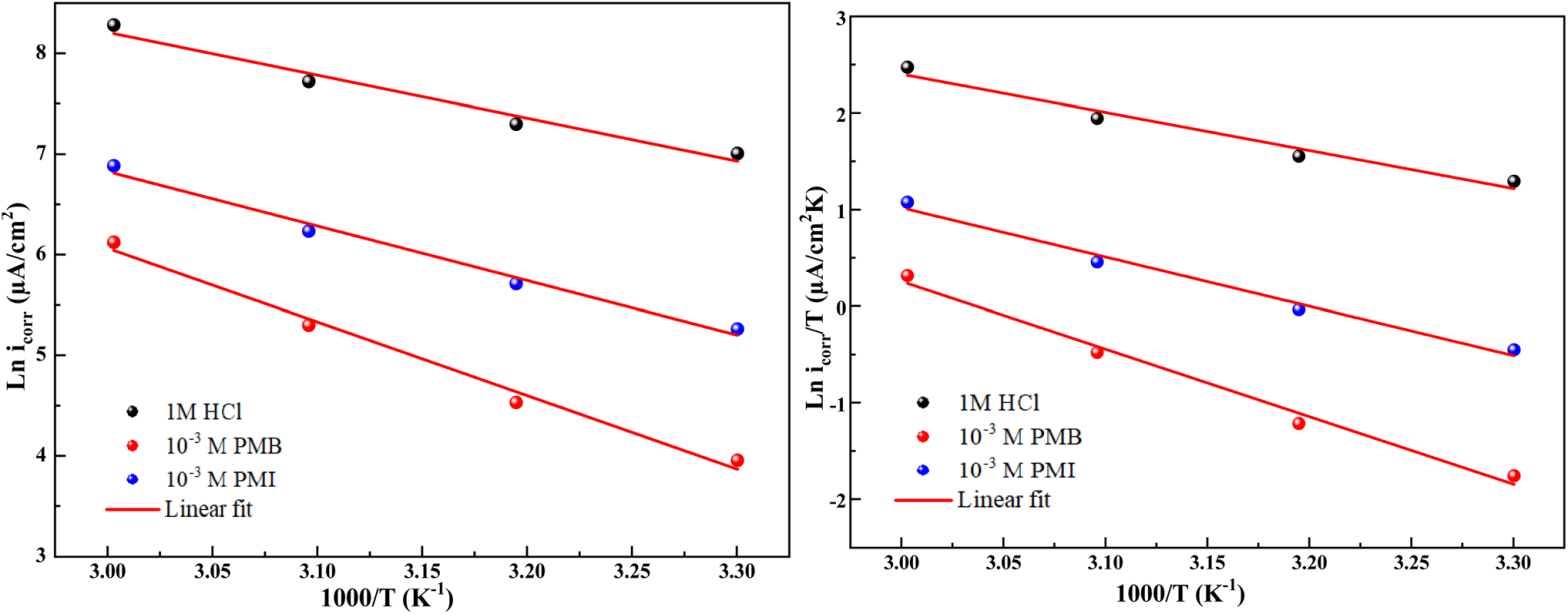
Arrhenius and transition state curves for CS corrosion in the existence and nonexistence of PMB and PMI inhibitors.

The Arrhenius and transition state diagrams for CS immersed in a 1 M HCl medium without and with optimum concentrations of PMB and PMI inhibitors at varying temperatures are shown in Fig. 5. These plots allow the effect of inhibitors on the kinetic parameters associated with the corrosion process to be studied as a function of temperature.

The Arrhenius and the transition state equations, whose mathematical expressions are given below, were used to investigate the relationship between temperature and corrosion rate.^9,34^ By linking the thermodynamic and kinetic parameters, these equations show how inhibitors affect the activation structure of the systems under investigation and the energy required to initiate the corrosion process.8
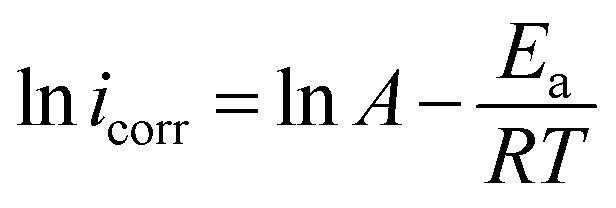
9
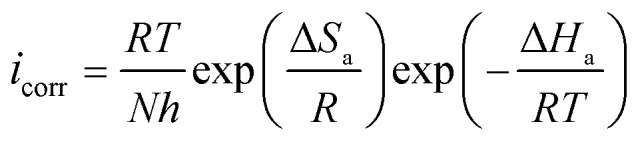


The corresponding activation parameters were determined and were tabulated in Table 3 by the slopes and intercepts of straight-line equations. These parameters include the activation enthalpy (Δ*H*_a_), the activation entropy (Δ*S*_a_), the pre-exponential factor, and the apparent activation energy (*E*_a_). These values are essential and informative concerning the mechanism of corrosion, with or without the inhibitors.

The activation parameters for the above-mentioned processes were calculated and listed in Table 4 from linear fits of the data according to relevant equations. The activation energy (*E*_a_), the activation enthalpy (Δ*H*_a_), and the activation entropy (Δ*S*_a_) were some of these calculated parameters. These parameters also give a general insight into corrosion kinetics and/or the existence or non-existence of the investigated inhibitors.

**Table 4:** Activation parameters for CS dissolution in 1 M HCl, both with and without 10^−3^ M PMB and PMI

	*E*_a_ (kJ mol^−1^)	Δ*H*_a_ (kJ mol^−1^)	Δ*S*_a_ (J mol^−1^ K^−1^)
1 M HCl	35.40	32.76	−79.18
PMI	45.02	42.38	−61.83
PMB	60.80	58.17	4.20

The findings in the table show that the inhibitors tested increased the energy barrier (*E*_a_) of the inhibited medium over that of the uninhibited medium. This rise in activation energy indicates how well the inhibition process was working, most likely as a result of the inhibitor molecules gradually adhering to the CS surface to form a protective film.

The dissolution process of carbon steel is endothermic,^35,36^ as indicated by the positive activation enthalpies (Δ*H*_a_). In general, a decrease in the dissolution rate of the metal is indicated by a rise in Δ*H*_a_ when the two inhibitors are present. In addition, the activation entropy (Δ*S*_a_) increases in the presence of the inhibitors compared to the uninhibited acidic solution. This increase supports the idea that a dynamic adsorption process favors the creation of a protective layer by indicating that the Fe–inhibitor complex formed on the surface is less organized.

### 3.3. EIS plots

**Fig. 6:**
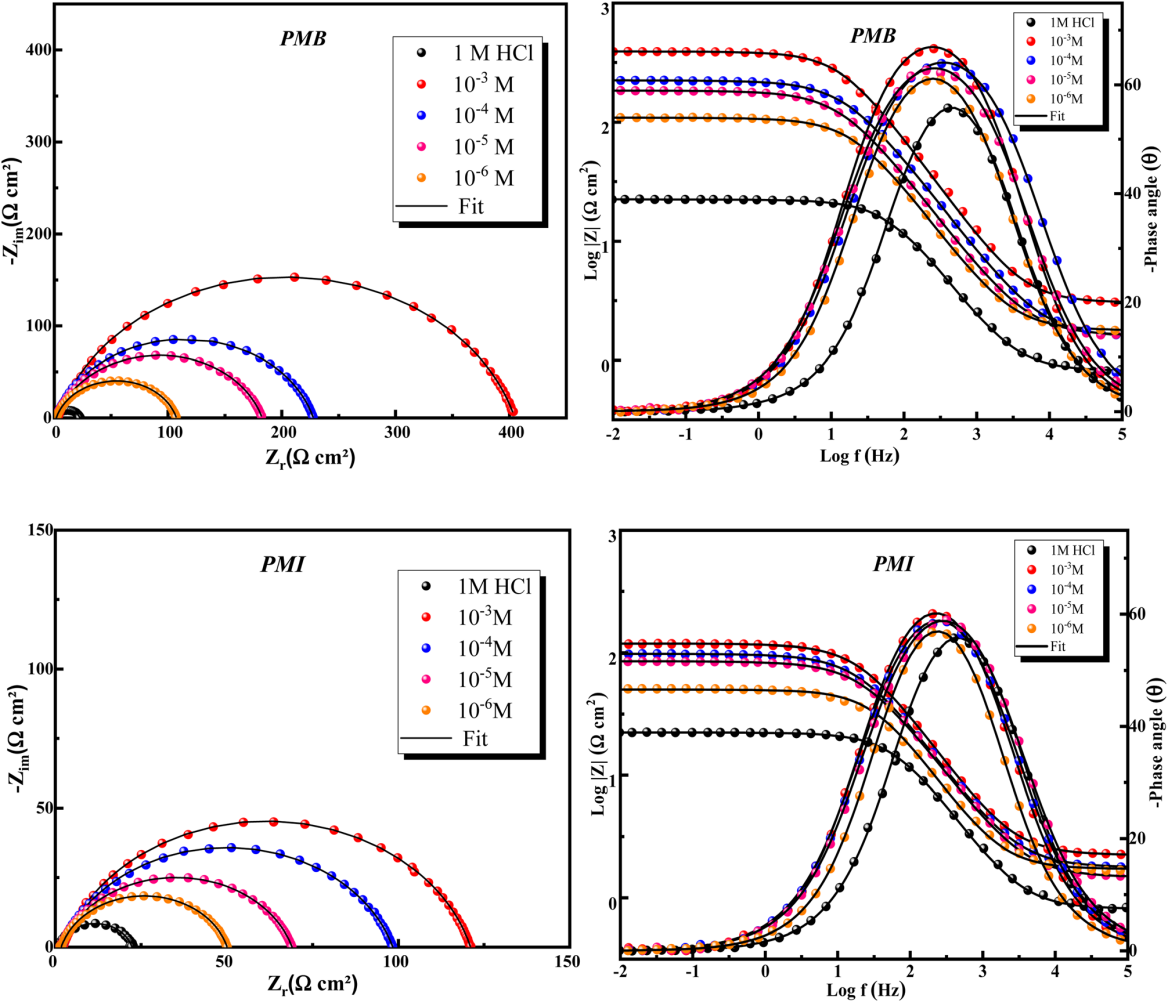
EIS and Bode plots for CS in 1 M HCl in the existence and nonexistence of different concentrations of PMB and PMI.

Electrochemical impedance spectroscopy (EIS) is an important method for examining inhibitors' interfacial characteristics and adsorption behavior. Fig. 6 shows the Nyquist and Bode curves for CS in 1 M HCl in the existence and absence of the two pyrazole derivatives PMB and PMI.

Fig. 6 illustrates how the inhibitor concentration affects the impedance behavior of CS in a 1 M HCl medium at 303 K. In the presence of varying PMB and PMI concentrations, the Nyquist curves for CS show a similar pattern. The center of the small semicircle represented by these curves lies below the real axis. The size of this semicircle rises with inhibitor concentration, suggesting that charge transfer is principally responsible for regulating the corrosion process.^37,38^ The imperfect semi-circular shape is an indication of a frequency dispersion commonly correlated to roughness and defects on the metallic surface. The presence of inhibitors, PMB and PMI, caused a significant change in the electrochemical impedance behavior of CS due to their effect on the corrosion mechanism.

Data in Table 5 show that the addition of PMB and PMI effectively protects CS in 1 M HCl solution. The higher value of *R*_p_ with increasing concentration indicates that these compounds inhibit corrosion, and the effectiveness of inhibition increases with the inhibitor concentration.

**Table 5:** EIS data of CS in 1 M HCl in the existence and non-existence of PMB and PMI

	Conc. (M)	*R*_s_ (Ω cm^2^)	*R*_p_ (Ω cm^2^)	10^6^ × *A* (Ω^−1^ s^*n*−1^ cm^−2^)	*n*_dl_	*C*_dl_ (µF cm^−2^)	*χ* ^2^	*θ*	*η*_EIS_%
HCl	1 M	0.8	21.6 ± 0.6	293.90	0.845	116.20	0.002	—	—
PMB	10^−3^	1.7	401.6 ± 2.1	64.8	0.832	30.7	0.007	0.946	**94.6**
	10^−4^	1.6	226.0 ± 1.8	90.8	0.834	41.0	0.008	0.904	90.4
	10^−5^	1.8	181.1 ± 1.3	132.8	0.821	58.6	0.009	0.881	88.1
	10^−6^	1.8	105.5 ± 4.0	163.0	0.837	70.9	0.009	0.795	79.5
PMI	10^−3^	1.7	118.8 ± 1.3	146.5	0.832	63.9	0.009	0.818	**81.8**
	10^−4^	1.6	96.6 ± 3.2	197.7	0.811	78.1	0.006	0.776	77.6
	10^−5^	1.7	67.5 ± 1.7	216.1	0.814	80.1	0.006	0.680	68.0
	10^−6^	2.0	48.4 ± 1.0	260.0	0.823	99.5	0.004	0.554	55.4

The equation below was utilized to calculate the inhibition effectiveness (*η*_EIS_) from the charge transfer resistance:^39^10
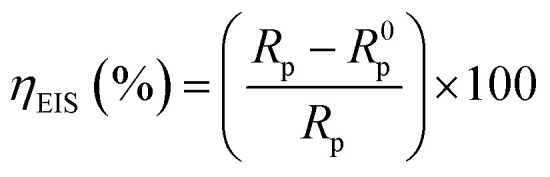
where *R*_p_ and *R*^0^_p_ denotes respectively, the resistance polarization in the existence and nonexistence of PMB and PMI.

**Fig. 7:**
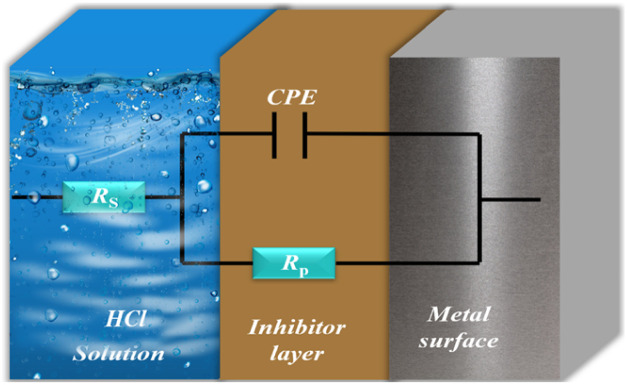
The equivalent circuit.

A simple equivalent circuit was utilized to examine the impedance spectrum representing a single capacitive semicircle (Fig. 7). This circuit consists of three basic components: a solution resistance (*R*_s_), a constant phase element (CPE), and a polarization resistor (*R*_p_).

The resistor (*R*_s_) in this model is connected in series with the polarization resistor (*R*_p_) and the CPE in parallel. The resistance associated with charge transfer and polarization processes at the metal/solution interface is represented by (*R*_p_), while the non-ideal capacitance of the electrochemical double layer is represented by the constant phase element (CPE).

The double-layer capacitance (*C*_dl_) was calculated using the relationship below:^40,41^11
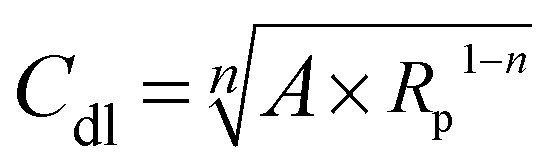


Furthermore, the rise in the phase angle value with increasing inhibitor concentrations shows strong adsorption of pyrazole derivatives onto the CS surface. The Bode plots show that the addition of PMB and PMI significantly increases the logarithmic amplitudes (log|*Z*|) compared to the 1 M HCl solution without inhibitors, demonstrating these inhibitors' remarkable corrosion protection capabilities.

Table 5 indicates that when PMB and PMI inhibitors are present, the polarization resistance (*R*_p_) rises noticeably in comparison to the blank electrolyte. This rise can be explained by the carbon steel's surface developing a protective layer that stops more corrosion. There is a clear correlation between better inhibition efficiency (*η*_EIS_%) and higher polarization resistance. As a result, the PMB and PMI inhibitors exhibit the highest inhibitory efficiency, with PMB reaching 94.6% and PMI reaching 81.8% at 10^−3^ M. With PMB providing the highest inhibition capability, our results validate the two inhibitors' outstanding effectiveness. At the same time, the capacity of the electrical double layer (*C*_dl_) decreased as the inhibitor concentration increased. As a direct result of the inhibitor molecules adhering to the metal surface, the thickness of the electrical double layer gradually increases, causing this decrease.^42^ In addition, the reduction in the value of *n* suggests that the adsorption of the inhibitor causes an increase in the surface roughness. The fact that the charge transfer process is still the primary mechanism controlling the electrochemical reaction is reflected in this uneven roughness. Additionally, the simulated and experimental electrochemical impedance spectra (EIS) exhibit low error factor (*χ*^2^) values and perfect superposition. This demonstrates that the equivalent electrical circuit, which consists of the metal substrate, the corrosive media, and the corrosion inhibitor, is a useful tool for precisely describing the system being studied.

### 3.4. Adsorption isotherms

**Fig. 8:**
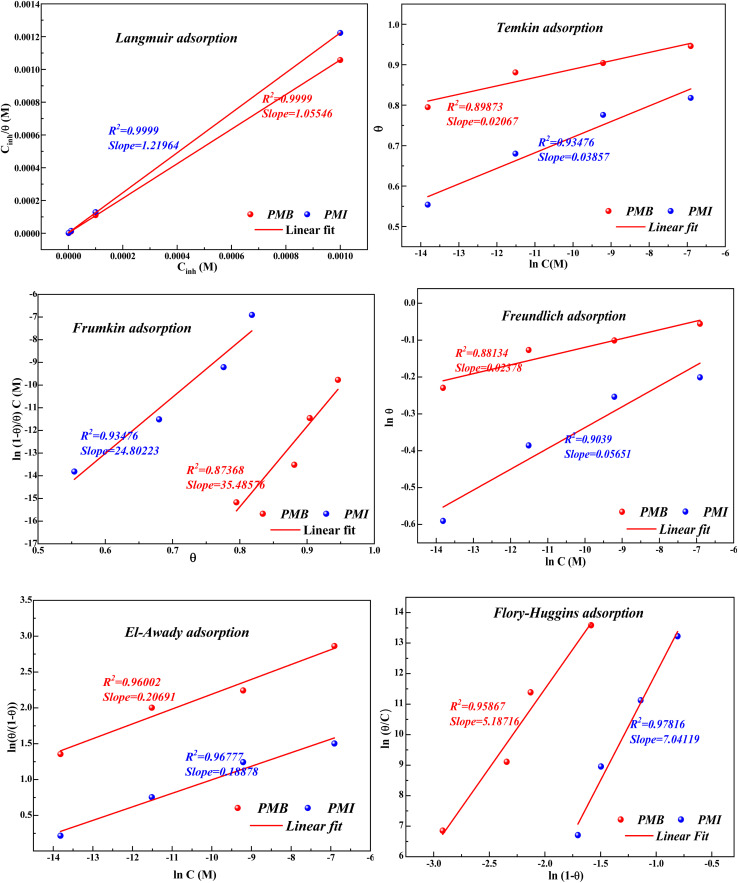
Adsorption isotherms for CS in 1 M HCl in the presence of PMB and PMI at 303 K.

The adsorption isotherm provides important details on the interaction of the inhibitor chemicals with the CS surface. The adsorption performance of the pyrazole derivatives PMB and PMI was proven to be best characterized by the Langmuir adsorption isotherm of all the isotherm models investigated, including Temkin, El-Awady, Frumkin, and Flory–Huggins (Fig. 8). The strong linearity of the experimental fits (*R*^2^ = 0.9999) and certain equations of this isotherm show that the adsorption of these inhibitors follows the principles of the Langmuir model.

The data show that the calculated adsorption constants *K* for PMB and PMI are 519 942.4 M^−1^ and 319 989.8 M^−1^, respectively. These high values demonstrate that the adsorption on the surface of the CS is highly efficient. In general, a high *K* value indicates a high adsorption capacity, which enhances the anti-corrosion performance of the inhibitors. In addition, the Δ*G*_ads_ values allow for evaluating the type of adsorption mechanism: physisorption is characterized by values around −20 kJ mol^−1^, whereas chemisorption is indicated by values below −40 kJ mol^−1^.^43,44^ The Δ*G*_ad_ values of the two inhibitors (Table 6), on the order of −42 kJ mol^−1^, indicate, consistent with what is generally reported in the literature, that the adsorption process is chemical in nature (chemisorption). However, it remains impossible to conclude with certainty whether the adsorption of the inhibitor molecules onto the metal surface is an exclusively chemical or exclusively physical phenomenon. Given that these values are close to the threshold of −40 kJ mol^−1^, it is reasonable to consider that the adsorption exhibits a mixed character, involving significant contributions from both physisorption and chemisorption.

**Table 6:** Adsorption parameters of PMB and PMI on the CS surface at 303 K

Inh.	Slope	*R* ^2^	*K* (L mol^−1^)	Δ*G*_ads_ (kJ mol^−1^)
PMB	1.05546	0.9999	519 942.4	−43.3
PMI	1.21964	0.9999	319 989.8	−42.0

### 3.5. Surface analysis

#### 3.5.1. UV-vis

**Fig. 9:**
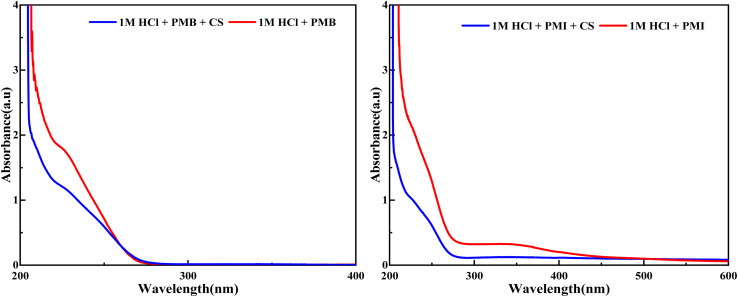
UV-visible spectra of PMB and PMI before and after dipping in 1 M HCl solution.

The UV-visible spectrum shows the absorbance as a function of wavelength in an acid solution (HCl) containing the PMB and PMI inhibitors, with or without steel (Fig. 9). In HCl alone, the absorbance of the inhibitors is much lower, which means that they are well dissolved and do not interact among themselves. On the other hand, the absorbance at this wavelength of the HCl solution with PMB and PMI inhibitors in the steel condition is greater than that of the steel-free condition, indicating an interaction between such inhibitors and metal ions that are produced by corrosion of steel, mainly Fe^2+^ and Fe^3+^. This increase in absorbance can be attributed to a variety of interactions, including the formation of coordination complexes between the inhibitors and these ions, where functional groups present in the PMB and PMI inhibitors, such as amines, oxygens, and hydroxyls, play a key role as electron donors. In addition, electrostatic interactions between the inhibitor charges and ionic species in solution are likely, as well as physical adsorption *via* van der Waals forces or chemical adsorption *via* covalent bonding to the metal surface. All these interactions indicate that the inhibitors actively modify the corrosion process by forming a protective layer on the steel surface.

#### 3.5.2. Contact angle analysis

**Fig. 10:**
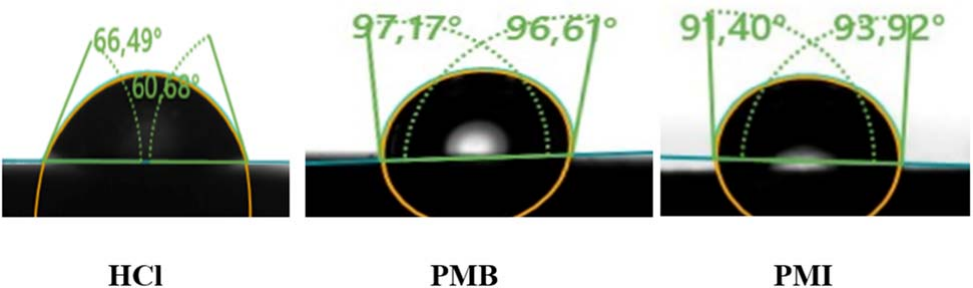
Contact angle for carbon steel in 1 M HCl solution without and with the presence of PMB and PMI inhibitors.

Fig. 10 shows the contact angles of the blank solution (a) in the presence of PMB (b) and PMI (c) at 6 hours as immersion time. The study showed that the initial surface of CS immersed in 1 M HCl medium was hydrophilic with a contact angle of 63.58°. This hydrophilicity favors direct contact with the corrosive media and can accelerate the corrosion process. However, after adding the PMB and PMI inhibitors, the contact angle increased significantly to 96.89° and 92.66°, respectively. This increase represents a shift in the surface towards a hydrophobic nature, which means that water wettability decreases. This improved hydrophobicity is mainly due to the creation of a protective layer adsorbed on the metal, which acts as a physical barrier against contact with the aggressive aquatic environment.^45,46^ In addition, inhibitor PMB imparts a higher degree of hydrophobicity than PMI, which is compatible with the experimentally determined order of corrosion inhibition effectiveness.

#### 3.5.3. SEM/EDS

**Fig. 11:**
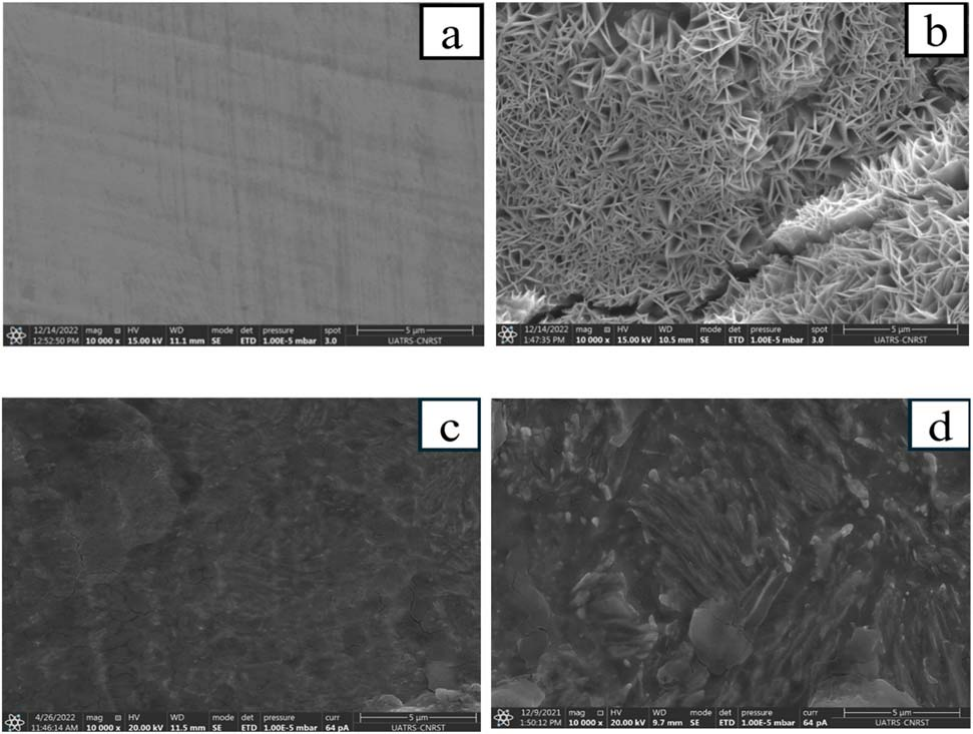
MEB images for CS only (a), in 1 M HCl solution (b), in the presence of PMB (c), and PMI (d) inhibitors at 303 K.

**Fig. 12:**
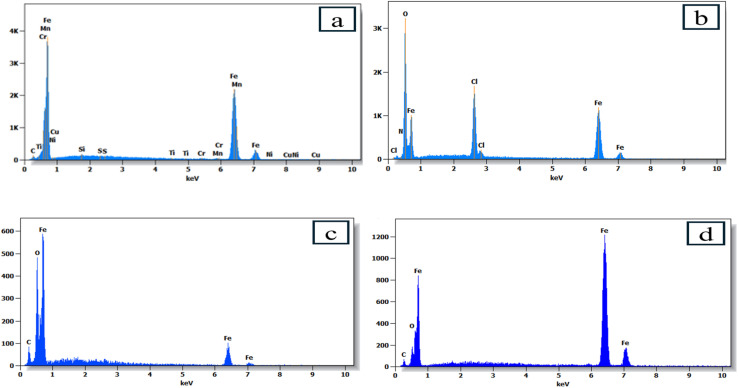
EDS spectra for CS only (a), in 1 M HCl solution (b), in the presence of PMB (c), and PMI (d) inhibitors at 303 K.

The images of carbon steel immersed in 1 M HCl solution for 24 hours show marked differences between the samples treated with PMB and PMI inhibitors and those without, as shown in Fig. 11. The image of the polished steel shows a uniform surface, whereas the image of the corroded steel without inhibitors shows significant degradation characterized by the thickness of corrosion products. In comparison, the samples treated with PMB and PMI inhibitors display a smooth surface due to the good corrosion protection provided by a protective film. This layer minimizes the interface between the metal and the acid solution, decreasing damage from corrosive ions. The findings highlight the efficiency of these compounds, PMB and PMI, in protecting carbon steel. In addition, the composition of metal surfaces was determined by EDS spectra. Fig. 12 indicates that the Cl^−^ ion peak disappears in the presence of inhibitors. Moreover, the decrease in the height of iron peaks associated with inhibitor molecules demonstrates that these organic compounds could be adsorbed on the metal surface and complexed to it as a thin film, which will prevent chloride ions from reaching the metal.

### 3.6. DFT

**Fig. 13:**
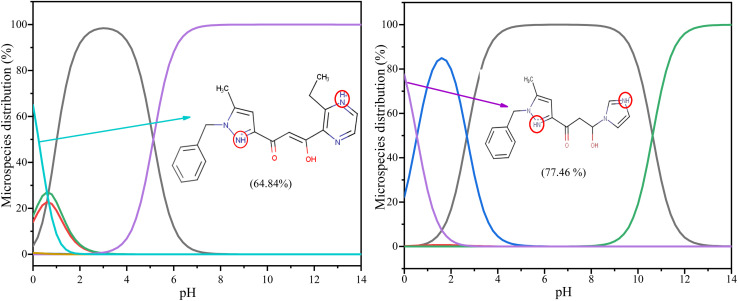
Protonation percentage *versus* pH of PMB and PMI tested using the MarvinSketch program.

The DFT computations were used in aqueous solutions to interpret experimental results and correlate inhibitory efficiency with the structure of an inhibitor. Molecular descriptors were calculated with a density functional theory (DFT)/B3LYP method at the base level 6-311G(d,p). In an acidic medium, the organic molecules under investigation tend to be protonated. This tendency is accentuated by the presence of heteroatoms, which affect the distribution of protonated species in relation to pH. The distribution percentages of the various species were evaluated using MarvinSketch software, which also identified the most probable sites of protonation in an acidic environment. Fig. 13 indicates that the molecules have major protonation at pH = 0, with 64.84% for PMB and 77.46% for PMI. Additionally, by modifying the acid medium's conditions, this study enables the optimization of the percentage distribution of the various protonated forms in the electrolyte.

**Fig. 14:**
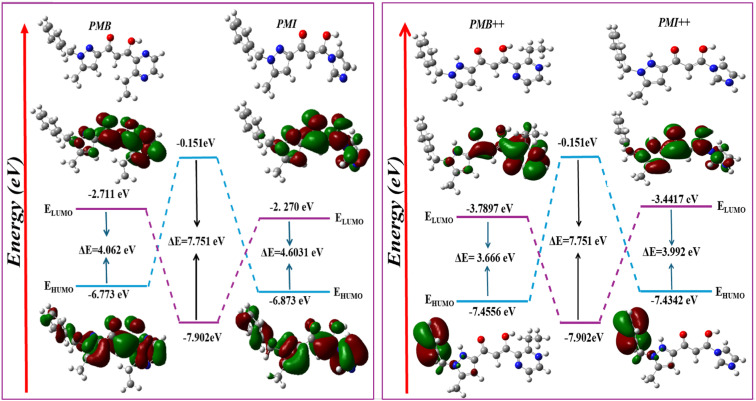
HOMOs, LUMOs, and the energy gap diagram of the neutral and protonated molecules under study using the B3LYP/6-31+G(d,p) level in aqueous medium.

Fig. 14 shows the electron density distributions of the frontier molecular orbitals (FMOs) for the neutral and protonated forms of PMB and PMI in their ground states. This figure demonstrates that the HOMO frontier orbitals of both molecule types are evenly distributed throughout the structure. This suggests that these molecules act as electron donors, allowing electrons to move to unoccupied d-orbitals on the surface of the ferrous metal. The electronic distribution of the LUMO orbitals covers virtually the entire molecular structure, except the phenyl groups. This property shows that these molecules have both electron donor and acceptor sites, which enhances their ability to adsorb on the metal surface.

To investigate the inhibitory mechanism of the two compounds investigated *via* their electronic characteristics, numerous quantum chemical descriptors for the neutral and protonated forms were determined. Table 7 shows the values for the energy of the most occupied molecular orbital (*E*_HOMO_), the energy of the lowest unoccupied molecular orbital (*E*_LUMO_), the energy band gap (*E*_gap_), the electronegativity (*χ*), the overall hardness (*η*), the electrophilicity (*ω*), and the nucleophilicity (*ε*) index of PMB and PMI.

**Table 7:** The quantum chemical descriptors for the neutral and protonated forms of PMB and PMI inhibitors

Parameters	PMB	PMI	PMB^++^	PMI^++^
*E*_HOMO_ (eV)	−6.773	−6.873	−7.456	−7.434
*E*_LUMO_ (eV)	−2.711	−2.270	−3.789	−3.442
*I* (eV)	6.773	6.873	7.456	7.434
*A* (eV)	2.711	2.270	3.789	3.442
Δ*E* (eV)	4.062	4.603	3.666	3.992
*χ* (eV)	4.742	4.572	5.623	5.438
*η* (eV)	2.031	2.302	1.834	1.996
*ω* (eV)	5.536	4.541	8.621	7.408
*ε* (eV)	0.181	0.220	0.116	0.135
Δ*N*_110_	0.019	0.054	−0.219	−0.155

Table 7 shows the various quantum chemical descriptors derived at the B3LYP level for the PMB and PMI molecules under investigation. *E*_HOMO_ values indicate the ability of a molecule to lose electrons, whereas a low *E*_LUMO_ value suggests an ability to accept electrons. Thus, the higher the *E*_HOMO_, the more likely inhibitors will transfer electrons to species with unoccupied molecular orbitals and lower energy levels. In this study, the *E*_HOMO_ values for PMB and PMI are −6.773 eV and −6.873 eV, respectively, showing that the PMB molecule provides more electrons than PMI. This pattern is consistent with the order of inhibition efficiency, with PMB being a more potent inhibitor than PMI. In reality, the inhibition potential increases with the value of *E*_HOMO_, with the highest value for PMB indicating its maximum electron-donating capability.^47^ In addition, the energy gap (Δ*E*) indicates the interaction between the PMB and PMI inhibitors and the iron metal surface. In general, lower energy gaps indicate better and more persistent inhibitors. The energy gap between PMB and PMI is relatively small, 4.062 eV for PMB and 4.603 eV for PMI. In terms of stability and reactivity, PMB outperforms PMI. In addition, a higher *χ* usually accompanies a high level of inhibition performance.^48^Table 7 shows that the *χ* values are in the order of PMB (4.742 eV) > PMI (4.572 eV), and this is consistent with the experiment. Furthermore, those inhibitors with higher hardness (*η*) may influence their adsorption on metal surfaces. The hardness (*η*) of the compounds is inversely correlated with their polarizability. A smaller *η* value leads to a molecule that is more polarizable and consequently more probable to interact with the metal. The reported values (*η* = 2.031 eV for PMB and *η* = 2.302 eV for PMI) reveal that PMB is slightly less than PMI, suggesting the enhanced tendency to release electrons and extensive interaction with the metal surface.^49^ Electrophilicity (*ω*) is a parameter that measures the tendency of a molecule to accept electrons, and nucleophilicity (*ε*) measures its tendency to donate electrons.^50,51^ A good nucleophile generally has a low *ω* and high *ε* value, while a strong electrophile will have a high *ω* and low *ε*. Based on Table 7, PMB is less electrophilic and more nucleophilic than PMI, the former being able to inhibit corrosion better than PMI. The percentage of electron transfer (Δ*N*_110_) enables the evaluation of the ability of inhibitor molecules to donate electrons and constitutes a determining factor in their corrosion inhibition efficiency. A lower Δ*N*_110_ value (less than 3.6) indicates a more effective inhibitor, as it reflects a greater propensity for electron donation to the metal surface.^52^ The results obtained in the present study show that Δ*N*_110_(PMI) > Δ*N*_110_(PMB), suggesting that PMB exhibits a higher tendency to donate electrons to iron. Consequently, PMB can be considered a more efficient corrosion inhibitor than PMI. This finding is in good agreement with the other electronic parameters and experimental results, further confirming the superior corrosion protection performance of PMB compared to PMI.

Quantum chemical descriptors indicate that the energy gap (*E*_gap_) is lower for the protonated species than for their neutral counterparts. This reduced *E*_gap_ clearly reflects the higher reactivity of the protonated forms and their stronger interaction with the metal surface. Consequently, the protonated molecules are more readily adsorbed onto carbon steel compared to their neutral analogues. This behavior can be attributed to the increased polarity and enhanced electron-transfer capability of the protonated species, which promotes stronger interactions with the metal surface and leads to improved corrosion inhibition efficiency. Furthermore, the reactivity order of the protonated species is consistent with that of their corresponding neutral forms, indicating that protonation does not alter the relative inhibition tendency. Based on these findings, PMB exhibits a higher adsorption capacity and superior inhibition efficiency compared to PMI in both neutral and protonated media.

#### 3.6.1. Molecular electrostatic potential (MEP)

**Fig. 15:**
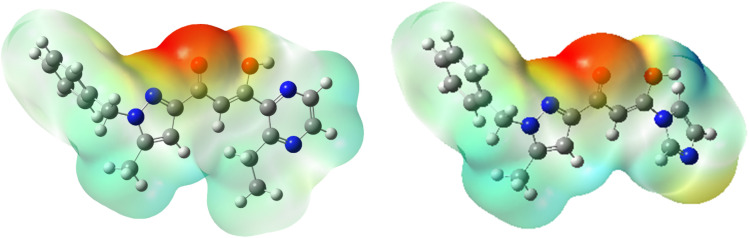
Molecular electrostatic potential (MEP) of the PMB and PMI molecules.

Fig. 15 illustrates the molecular electrostatic potential surfaces (MEPs) of the two investigated inhibitors, PMB and PMI, which are used to identify regions of electron density. These potentials are mapped using a color scale to visualize the charge distribution over the molecular surfaces. According to this scale, the regions of decreasing electrostatic potential follow the order: red > orange > yellow > green > blue.^53–55^ In the two molecules analyzed, areas of high electron density (from yellow to red) are mainly found on oxygen and nitrogen atoms, while areas of low electron density (from green to blue) are found on certain carbon atoms. The PEM can also be used to interpret the chemical reactivity of the molecules: negative areas (red and yellow) indicate electrophilic reactivity, and positive areas (blue) indicate nucleophilic reactivity. In our case, the study shows that the most negative area is around the oxygen atoms. Finally, Fig. 15 shows that the electrophilic zones, indicated in blue, cover a considerable part of the molecular surface of the two inhibitors, indicating a low electron density and a low tendency to lose electrons.

#### 3.6.2. Fukui functions

**Fig. 16:**
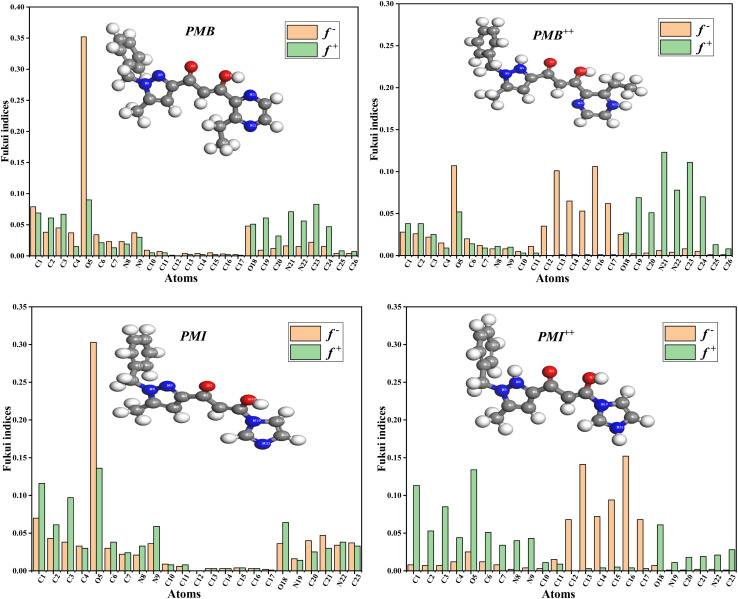
Fukui indices for PMB, PMI, PMB^++^, and PMI^++^.

The local reactivity of the inhibitors was assessed using the Fukui indices, which indicate the reactive areas and the nucleophilic and electrophilic behavior of inhibitor molecules. Fig. 16 shows the computed Fukui indices for two inhibitors, PMB and PMI, in both their natural and protonated forms.

The reactive sites for nucleophiles are indicated by the highest *f*^+^ indices in the PMB molecule, found at O5 (0.352). This analysis demonstrates that oxygen constitutes a significant site for nucleophilic attack and acts as a major electrophilic target. In addition, the carbon atoms C1 (0.079), C3 (0.045), and C4 (0.037) exhibit moderate to high *f*^+^ values, indicating their susceptibility to nucleophilic attack. Nucleophilic attack at the oxygen atom O18 (0.048) is also feasible. In contrast, C1 (0.069), C2 (0.061), C3 (0.067), O5 (0.090), O18 (0.051), and C23 (0.083) had the highest *f*^−^ indices, indicating electrophilic reactive sites. The most nucleophilic reactive sites for PMB^++^ are O5 (0.107), C13 (0.101), C16 (0.106), and C23 (0.111). This indicates that these atoms have a higher affinity for electrons and, consequently, a greater propensity to interact with nucleophiles. Regarding electrophilic attack sites, N21 (0.123), N22 (0.078), C19 (0.069), and C20 (0.051) have higher *f*^−^ indices values. N21 and N22 act as electron donors, making them more susceptible to electrophilic attack. These results suggest that the PMB molecule can be protonated and has well-defined nucleophilic and electrophilic sites that react differently depending on the type of chemical attack. The highest *f*^+^ values were observed at the O5 (0.303), C1 (0.070), C2 (0.043), and C21 (0.47) atoms for the PMI inhibitor. These atoms are the best electron acceptors, meaning they are the sites where nucleophiles are most likely to capture electrons. Furthermore, the *f* indices indicate both electrophilic and nucleophilic character for the best electron-donating sites. The highest values were observed for C4 (0.116), C2 (0.061), C3 (0.097), O5 (0.136), N9 (0.059), and O18 (0.064). This suggests that these are the preferred sites for electron donation to electrophiles, which is consistent with the experimental data. In PMI^++^, nucleophilic reactivity is characterized by high *f*^+^ indices on carbons C13 (0.141), C16 (0.152), and C15 (0.094), as well as C12 (0.068) and C14 (0.072), indicating that these sites are strong electron acceptors and likely to interact with nucleophiles. At the same time, C1 (0.113), C2 (0.053), C3 (0.085), C4 (0.044), O5 (0.134), and O18 (0.061) had the highest *f*^−^ indices, which are associated with sites that are reactive for electrophiles and electron donors. According to these results, electrophiles prefer to transfer electrons to oxygen and carbons close to functional groups.

### 3.7. MD simulation

**Fig. 17:**
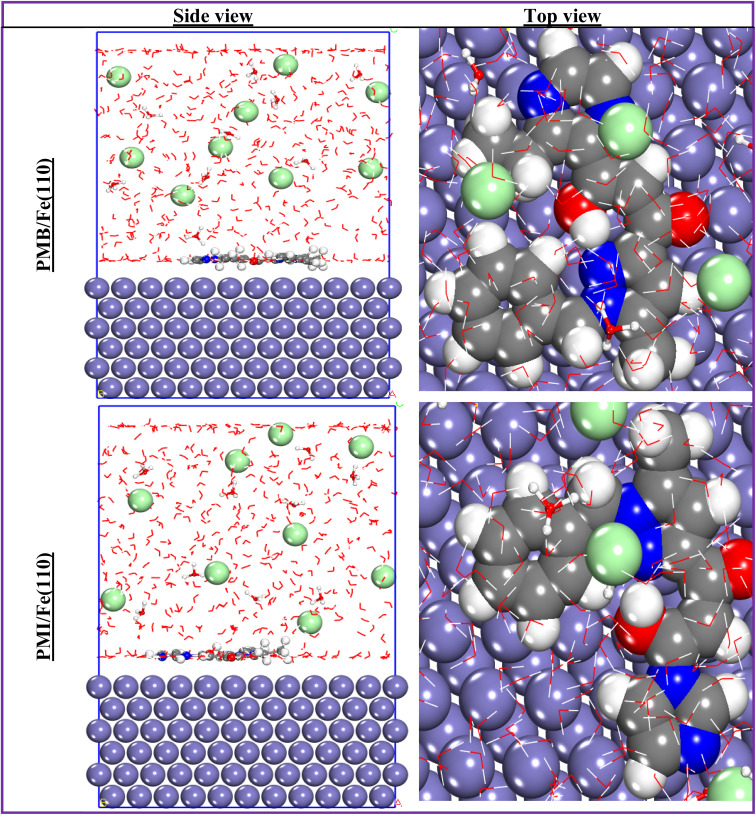
Best and most stable adsorption configurations of both PMB and PMI onto the Fe(110) surface.

**Fig. 18:**
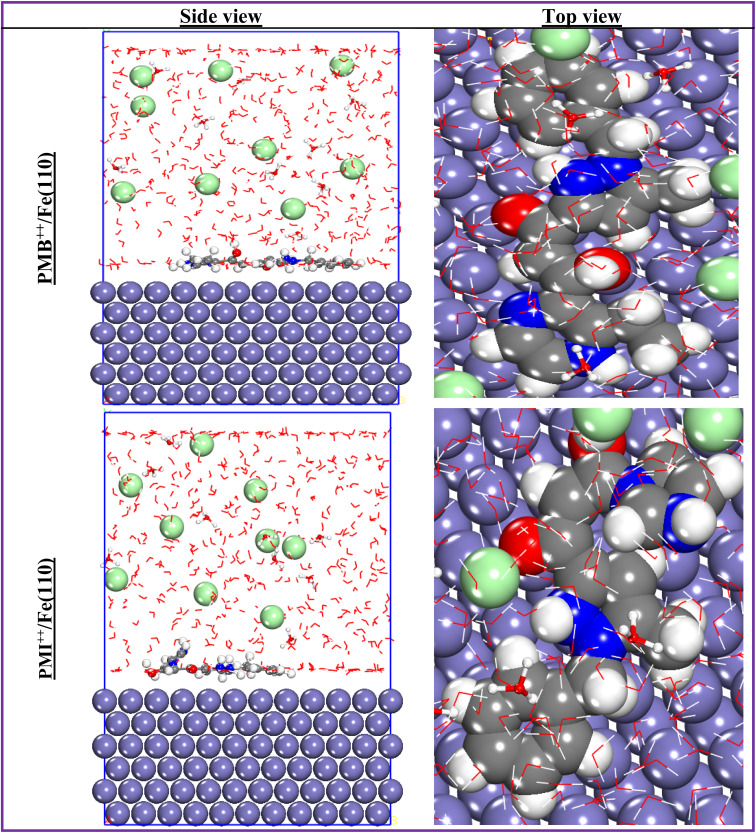
Most stable adsorption configurations of both PMB^++^ and PMI^++^ onto the Fe(110) surface.

The use of molecular dynamics simulation as an applicable approach to understanding the mechanism of inhibition of the two inhibitors investigated on the surface of the metal is very important. In this type of simulation, it is vital to take into account all the chemical species used in the experimental part.^56^ The use of MD allows us to evaluate and compare the inhibitory efficiency of the two molecules, PMB and PMI, to verify a possible correlation between the MD simulation data and the experimental inhibitory efficiency values. To this end, Fig. 17 and 18 display the most stable and favorable adsorption configurations of the PMB, PMI, PMB^++^, and PMI^++^ onto the ordered surface of Fe(110). In these figures, the inhibitor molecules align parallel across the first atomic layer of iron, suggesting effective adsorption over a large portion of the metal surface. This pattern of interaction reflects a strong affinity between the inhibitors and the metal substrate. This adsorption behavior thus supports the decisive role of the two inhibitors in protecting the metal surface, mainly through the formation of a stable, long-lasting protective coating.^56^

The adsorption configurations mainly represent a visual depiction of the interaction of the inhibitors studied with the Fe(110) surface. But in order to make a more precise and quantitative measurement of the degree of efficiency of the inhibitor–surface interaction, the introduction of an energy index is essential: the interaction energy (*E*_interaction_), calculated on an empirical scale. The literature suggests that a low negative value (high absolute value) of this index reflects a high capacity for interaction between the inhibitor molecule and the metal surface. This interaction energy is calculated using the following formula:^57^

12 *E*_interaction_ = *E*_total of system_ − *E*_surface+solution_ − *E*_inhibitor_

The calculated values of the interaction energy are collected in Table 8. It is observed that the PMB^++^/Fe(110) interaction system exhibits a low value of the interaction energy (−1472.882 kJ mol^−1^), revealing higher adsorption of PMB^++^ onto the Fe(110) surface. In addition, the negative value for all systems reflects a strong interaction between the compounds studied and Fe(110). In fact, these values testified to the existence of significant attractive forces between the species present, strengthening the stability of adsorption and confirming the effectiveness of the support as an anchoring site for both molecules tested.^27^

**Table 8:** *E*_interaction_ of four systems of PMB/Fe(110), PMI/Fe(110), PMB^++^/Fe(110), and PMI^++^/Fe(110) (kJ mol^−1^) in HCl

Systems	*E*_interaction_
PMB/Fe(110)	−1468.606
PMI/Fe(110)	−1399.587
PMB^++^/Fe(110)	−1472.882
PMI^++^/Fe(110)	−1305.529

**Fig. 19:**
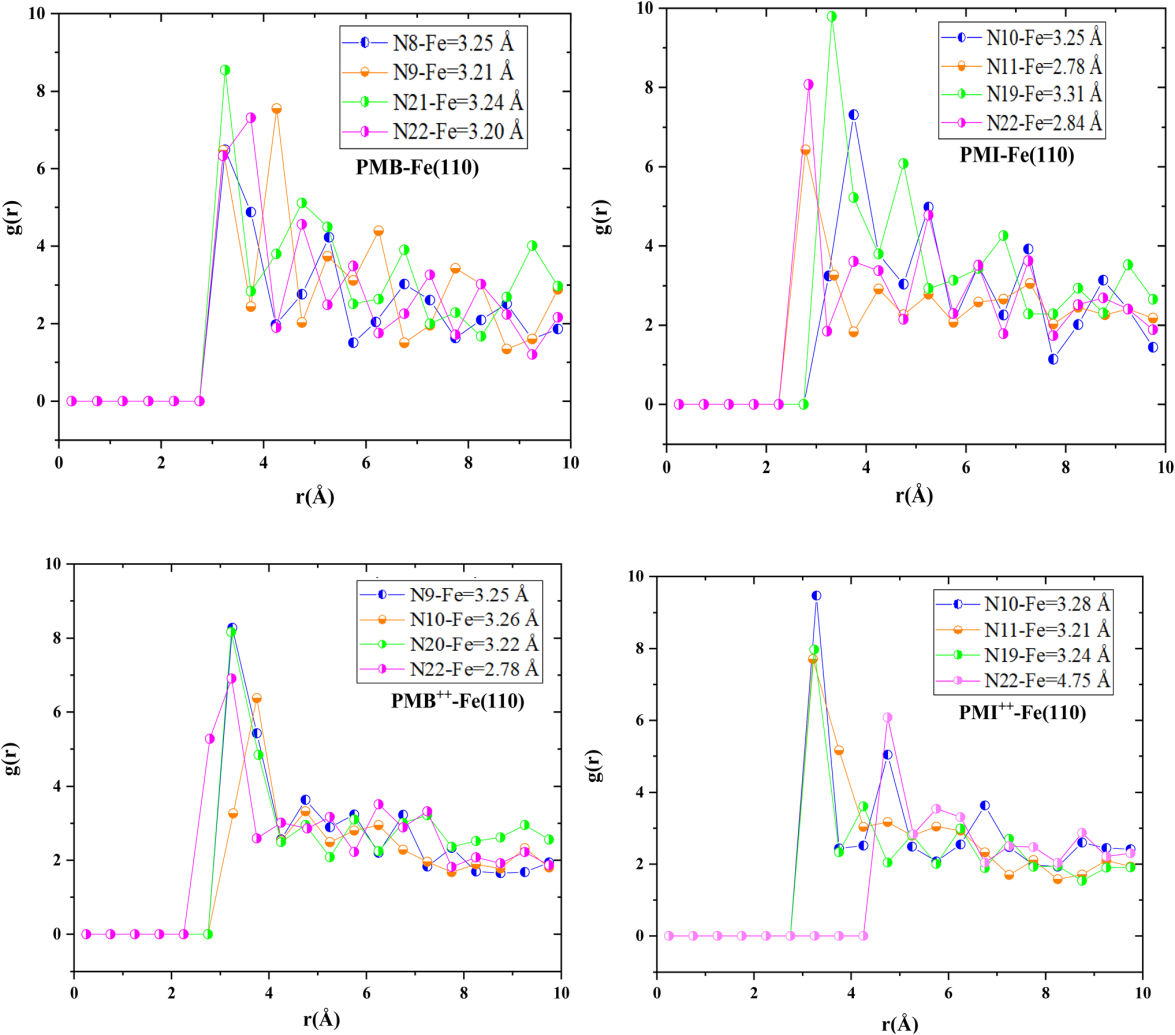
RDF of the PMB, PMI, PMB^++^, and PMI^++^ onto Fe(110).

This point is validated by analysis of the radial distribution function (RDF), depicted in Fig. 19, and in particular by the bond distances between Fe(110) and nitrogen atoms for the PMB, PMI, PMB^++^, and PMI^++^. The bond lengths recorded, mostly below 3.5 Å, suggest a strong chemical interaction between the inhibitors and the Fe(110) surface, typical of chemical adsorption. Conversely, a bond distance greater than 3.5 Å, as in the case of the N22–Fe pair, indicates a physical interaction, probably linked to the charge carried by the N22 atom of the PMI^++^ molecule.^58^ Chemical and physical interactions contribute significantly to the protection of the steel studied against corrosion by providing an effective protective barrier that limits the penetration of aggressive species in acidic HCl.^58^

### 3.8. Inhibition mechanism analysis

The inhibition effect of PMB and PMI on the corrosion of CS in 1 M HCl is due to the absorption of the molecules, both chemisorption and physisorption, and thus the resultant formation of a protective Fe–PMB, Fe–PMI layer on the carbon steel surface. In an acid medium, carbon steel is anodically dissolved: Fe → Fe^2+^ + 2e^−^ and cathodic hydrogen evolution:2H^+^ + 2e^−^ → H_2_.

**Fig. 20:**
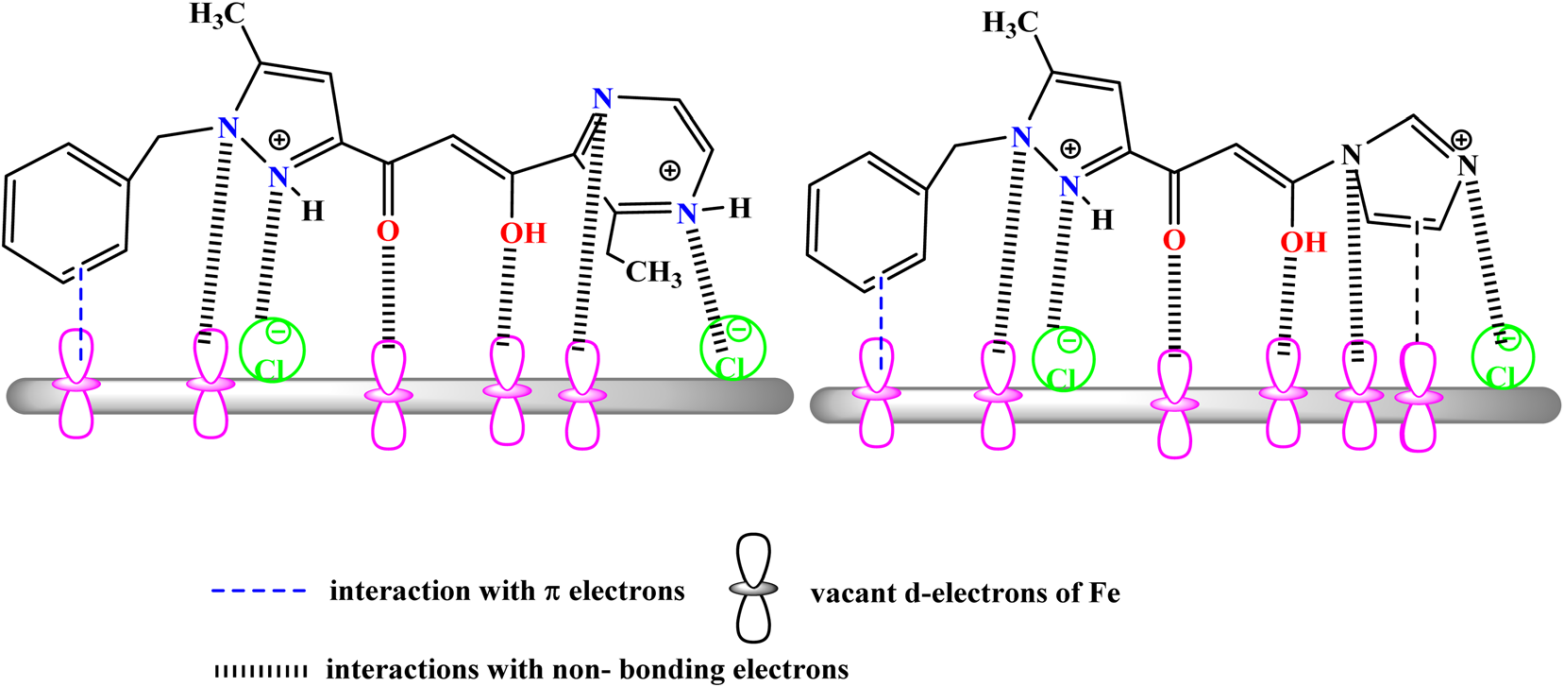
Corrosion inhibition mechanism for CS in 1 M HCl containing PMB and PMI.

However, the PMB and PMI addition break these processes. PMB and PMI molecules have a nitrogen atom rich in electrons and an aromatic π-electron system, which can chemically interact with the vacant d-orbitals of the iron atoms to form coordinate bonds with Fe–N, Fe–O, and Fe–π, firmly fixing the inhibitor strongly to the surface, as illustrated in Fig. 20.

In acid solution, PMB and PMI are present as neutral and protonated forms (nitrogen compounds): the neutral PMB and PMI are adsorbed firmly on the metal through electron donor–acceptor interactions, while the protonated PMB^++^ and PMI^++^ are attracted electrostatically to the negatively charged (chloride-adsorbed) steel surface, thus strengthening the surface physisorption and compactness of the film. This cooperative interaction results in the formation of a uniform, adherent, and hydrophobic barrier that blocks active sites, restricts the adsorption of H^+^ and Cl^−^ ions, and suppresses both anodic metal dissolution and cathodic hydrogen evolution. The enol, phenyl, imidazole, and pyrazine groups, present as substituents, increase electron density on the molecule, thus facilitating its adsorption affinity toward the steel surface. As a result, a robust protective layer is formed through a combination of Fe–inhibitor bonding, π–d-orbital interactions, and electrostatic forces, which restrict charge relocation and mass transport processes, thus providing an efficient barrier against the progression of corrosion.^59–61^

### 3.9. Environmental toxicity analysis

Environmental toxicity profiles of PMB and PMI determined with the Deep-PK tool have enlightened us with pretty good environmental safety of these chemicals. Prediction results of the Deep-PK tool are shown in Table 9. Both PMB and PMI are considered to be non-harmful to birds, as PBM and PMIs belong to the avian species safe category. Since they are biodegradable, the environmental persistence of these compounds is expected to be low. This will limit their accumulation in nature and consequently, their harmful effects. log *P* values of PMB and PMI are 3.374 and 2.676, respectively. The inhibitors possess sufficient hydrophobicity to be able to bioaccumulate in organisms and less susceptibility to leaching into water, but they are not so lipophilic as to be highly persistent or immobile. Very low BCF values for PMB and PMI (0.041 and 0.34, respectively) reflect the absence of bioaccumulation and biomagnification potential that makes them good candidates for the environment. There is moderate toxicity to *Daphnia magna* according to the LC_50_ value between 4.70 and 6.16 (log_10_[(mg L^−1^)/(1000 × MW)]); these species were more sensitive to the toxic compound, and the impact on *T. pyriformis* (−1.78 for PMB and 1.01 for PMI) suggests negligible microbial toxicity, indicating that basic aquatic microbial ecosystems may remain largely unaffected. Thus, from the chemical and environmental standpoints, PMB and PMI appear to be a good compromise of biodegradability and low environmental persistence with very minor ecological risks if properly handled.^62^

**Table 9:** Predicted toxicity profiles for PMB and PMI

Property name	PMB		PMI		Property unit
	Predicted value	Predictive confidence	Predicted value	Predictive confidence	
log *P*	3.374	—	2.676	—	log(mol L^−1^)
Avian	Safe	0.096	Safe	0.191	Category (toxic/safe)
Bioconcentration factor	0.041	—	0.34	—	log_10_(L kg^−1^)
Biodegradation	Safe	0.012	Safe	0.0	Category (safe/toxic)
*Daphnia magna*	4.70	—	6.16	—	−log_10_[(mg L^−1^)/(10^3^ × MW)]
*T. pyriformis*	−1.78	—	1.01	—	−log_10_[(mg L^−1^)/(10^3^ × MW)]

## 4. Conclusions

The inhibition effectiveness of two pyrazole derivatives, (*Z*)-1-(1-benzyl-5-methyl-1*H*-pyrazol-3-yl)-3-(3-ethylpyrazin-2-yl)-3-hydroxyprop-2-en-1-one (PMB) and (*Z*)-1-(1-benzyl-5-methyl-1*H*-pyrazol-3-yl)-3-hydroxy-3-(1*H*-imidazol-1-yl)prop-2-en-1-one (PMI), was assessed experimentally and theoretically. The following is a summary of the primary findings:

- The two pyrazole derivatives PMB and PMI were effective in preventing corrosion with inhibition efficacies of 95.3% and 82.5%, respectively.

- Both inhibitors showed a mixed-type inhibition behavior with *i*_corr_ decreasing in the following order: PMB (52.2 µA cm^−1^) > PMI (146.5 µA cm^−1^).

- EIS experiments revealed that the polarization resistance increased with the concentration of the inhibitors, implying that a protective barrier formed gradually when inhibitor molecules adsorbed on the surface of the metal.

- PMB and PMI adsorb on the surface of the carbon steel in accordance with the Langmuir isotherm, showing that both inhibitors adsorb *via* chemisorption.

- UV-visible spectroscopy confirms the formation of a complex inhibitor-fer.

- Surface analysis shows the creation of a protective coating on the carbon steel surface, which reduces the corrosive ions' attack.

- DFT and MD data show good agreement with experimental results.

- The replacement of the imidazole substituent (PMI) with an ethylpyrazin-2-yl group (PMB) introduces significant structural and electronic diversity due to differences in ring size, basicity, conjugation, and the presence of an ethyl spacer, which translates into distinct adsorption behaviors and corrosion inhibition efficiencies on steel.

## Conflicts of interest

There are no conflicts to declare.

## Data Availability

All data generated or analyzed during this study are included in this article.
